# Daily and Seasonal Activity Patterns of the Spiny-tailed Lizard (*Uromastyx aegyptia*) in Northern Saudi Arabia

**DOI:** 10.3390/life15050735

**Published:** 2025-05-01

**Authors:** Monif AlRashidi, Abdulaziz S. Alatawi, Sami Saeed M. Hassan, Mohammed Shobrak

**Affiliations:** 1 Department of Biology, Faculty of Science, University of Ha’il, Ha’il 55476, Saudi Arabia; 2 Department of Biology, Faculty of Science, University of Tabuk, Tabuk City 71491, Saudi Arabia; abalatawi@ut.edu.sa; 3 Department of Zoology, Faculty of Science, University of Khartoum, Khartoum 11115, Sudan; samisaeedmh@yahoo.com; 4 Terrestrial Wildlife Conservation Department, National Center for Wildlife, Riyadh 12411, Saudi Arabia; shobrak@saudibirds.org

**Keywords:** ectotherms, extreme environments, thermoregulatory behaviour

## Abstract

The Spiny-tailed Lizard (*Uromastyx aegyptia*), a vulnerable species native to the desert and semi-desert regions of the Middle East, remains poorly understood, particularly regarding its daily activity patterns in northern Saudi Arabia. This study, conducted in the Ha’il region, aimed to examine these patterns, assess the influence of soil temperature on activity, and identify potential threats to the species. The results revealed that soil temperature significantly affected the lizard’s activity patterns. During spring, Spiny-tailed Lizards were more active, spending around 25% of the day engaged in various behaviours, while their activity decreased to less than 20% in summer. In autumn and winter, the lizards did not follow a consistent daily activity, becoming active only when surface temperatures exceeded 35 °C. The absence of tracks and sightings in January suggests the species enters a state of complete brumation during this month. While no predation events were recorded via trail cameras, human disturbance from livestock and vehicles was observed in spring and summer. Although the disturbance was minor, reducing this type of human-caused disturbance should be taken into consideration when designing any protection programs. Furthermore, the long-term monitoring of this lizard’s daily and seasonal activity patterns is recommended in order to better understand its adaptability to environmental changes, especially those driven by climate fluctuations.

## 1. Introduction

Ambient temperature exerts a particularly strong influence on ectotherms, whose activity patterns are closely tied to thermal conditions [1,2,3,4]. Unlike endotherms, ectotherms rely on environmental heat for thermoregulation, necessitating active behavioural strategies—such as basking and shade-seeking—to maintain optimal body temperatures in the face of fluctuating thermal environments [5,6,7,8,9,10]. Understanding the factors that influence species’ activity patterns is therefore essential, as choosing the right activity window is critical for the survival of species such as lizards [11,12,13,14]. This is particularly important for species in arid environments, where adaptations are necessary in order to cope with extreme temperatures and limited food resources [15]. In the future, the suitability of microhabitats will likely be crucial for the survival of certain lizard species, enabling them to cope with climate fluctuations [16,17].

The Spiny-tailed Lizard (*Uromastyx aegyptia*) is a large terrestrial species that inhabits desert and semi-desert regions of the Middle East [1,5]. It is an opportunistic forager, primarily feeding on various plant parts, although insect remnants have also been found in its stomach and faecal pellets [1,18]. This species comprises three recognized subspecies: *Uromastyx aegyptia aegyptia*, *U. a. microlepis*, and *U. a. leptieni*. *U. a. aegyptia* is distributed from east of the Nile to Wadi Araba in Jordan, as well as Wadi Sawawin and the extreme northwestern region of Saudi Arabia. *U. a. microlepis* is widespread across Saudi Arabia, Oman, Yemen, the United Arab Emirates, Qatar, and Kuwait. In contrast, *U. a. leptieni* is restricted to the Hajar al-Gharbi Mountains in Oman and the northeastern United Arab Emirates [19,20]. Classified as vulnerable [20], this species faces significant pressure from anthropogenic activities across its natural range, resulting in a declining population trend [20,21]. Environmental changes linked to these disturbances may exceed the species’ tolerance capacity [22]. Climate warming is considered a potential survival threat for this species [5,20], highlighting the urgent need for conservation efforts [23].

The Spiny-tailed Lizard has been a subject of significant research due to its vulnerable status. Many studies across Saudi Arabia and the surrounding regions have examined various aspects of the species’ habitat selection, thermoregulation, activity patterns, food preferences, and feeding behaviour [1,5,14,22,24,25,26]. However, limited information exists regarding its daily and seasonal activity patterns, particularly in northern Saudi Arabia. To address this gap, the present study aims to quantify the daily and seasonal activity patterns of the Spiny-tailed Lizard, specifically the subspecies *U. a. microlepis*, in the Ha’il region. Additionally, the study examines the role of soil temperature as a key microclimatic factor influencing these activity patterns and documents potential anthropogenic or environmental disturbances that may pose threats to its northern population.

## 2. Materials and Methods

### 2.1. Study Area and Data Collection

**Figure 1:**
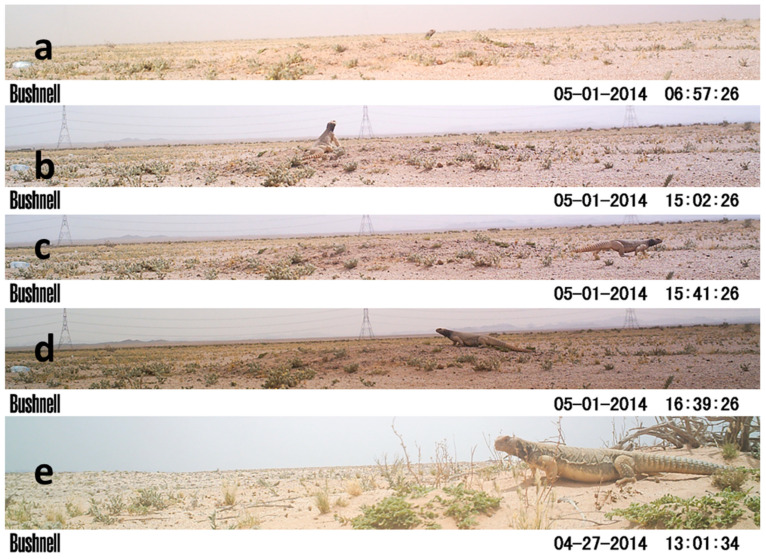
(**a**) Partial emergence, (**b**) basking, (**c**) leaving the burrow for foraging and other activities, (**d**) returning to the burrow, and (**e**) close-up view of the study species.

This study was conducted from 15 April 2014 to 10 February 2015. The study area is located approximately 30 km northeast of Ha’il city in Saudi Arabia (27.7076° N, 41.9196° E). It is a semi-arid to arid desert environment, typical of the central plateau of the Arabian Peninsula. The terrain is generally flat, interspersed with some valleys, and sparsely vegetated. Common plant species include halophytic shrubs (*Haloxylon salicornicum*) and the annual plant (*Arnebia hispidissima*) (Figure 1). The area is dotted with some scattered bushes of Arabian boxthorn (*Lycium shawii*) and a few acacia trees (*Vachellia gerrardi*).

A Bushnell 8 MP Trophy Cam Black LED Trail camera was used to monitor the daily activities of the Spiny-tailed Lizard and to capture any disturbances or threats near active burrows over 24 h. A total of 21 burrows were monitored: 13 during spring (11 in April and 2 in May) and 8 during summer (2 in June, 3 in July, and 3 in August). Nine cameras were deployed at active burrows for 24 h periods during autumn (September–November) and winter (December–February), but no activity was detected. As a result, the study focused on spring and summer. Each camera was positioned approximately 5 m from each burrow and set to capture an image every minute. It was equipped with infrared sensors, allowing it to record at night without emitting light or using a flash. To ensure minimal disruption, the camera was camouflaged with clay-coloured paint and was installed one day prior to the start of recording, allowing the lizards time to acclimate to its presence. Soil temperature was measured every minute near each burrow for at least 24 h using a HOBO U10 data logger, which was placed on the ground surface in an open area about 2 m from each burrow entrance. The distance between burrows ranged from 1 to 6 km, with a median of 3 km. The elevation of the studied burrows ranged from 859 to 976 m, with a median elevation of 928 m above sea level.

### 2.2. Data Analysis

Each day was divided into 24 h, and for each hour four behavioural parameters were analysed and calculated for each individual (Figure 1): (1) retreating underground (when a lizard stayed inside the burrow); (2) partial emergence (when the lizard peered its head only or its head and forelimbs out from the burrow entrance); (3) basking (when the lizard fully emerged from its burrow and exposed itself to the sun at or near the burrow entrance within the camera’s field of view); and (4) foraging and other activities (when the lizard ventured out its burrow to feed or engage in other activities, which was calculated from the moment it moved away from the burrow entrance until it returned). When the lizard ventured away from the burrow to feed outside the camera’s field of view, it might engage in additional activities such as basking, attracting a mate, or seeking shade elsewhere before returning to its burrow, all of which were categorised as ‘foraging and other activities’ behaviour.

A mixed-effects model was employed to examine the impact of soil temperature on daily activities and to determine whether there were significant differences in activity patterns between spring and summer. All response variables were transformed using ln(*x* + 1) to normalise the residual distributions. Burrow identity and hours were included as random factors. Initial models were fitted using the maximum likelihood method, and model selection was performed with the *stepAIC* function. The final model is reported after being refitted using Restricted Maximum Likelihood (REML). A paired *t*-test was used to assess whether there were significant differences in mean soil temperature between spring and summer. Statistical analyses and figure creation were performed using R version 4.3.3 [27]. Values are reported as mean ± SE.

## 3. Results

### 3.1. Seasonal Variations in Soil Temperature

**Figure 2:**
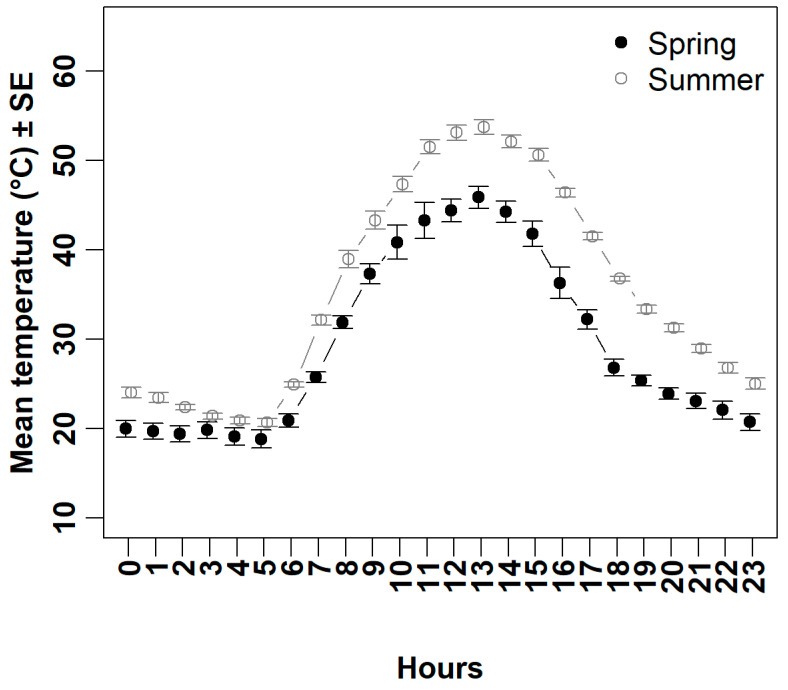
Variations in soil temperature between spring and summer (mean ± SE for each hour) in an open area approximately 2 m from the burrow entrance. Data were recorded over 21 days, with 13 days in spring and 8 days in summer.

Overall, the variations in soil temperature between spring and summer were significant (Figure 2). The mean soil temperatures were substantially higher in summer compared to spring (paired t = 23.36, *df* = 191, *p* < 0.01), with the difference being particularly pronounced during the hottest parts of the day (Figure 2).

### 3.2. Retreating Underground

**Figure 3:**
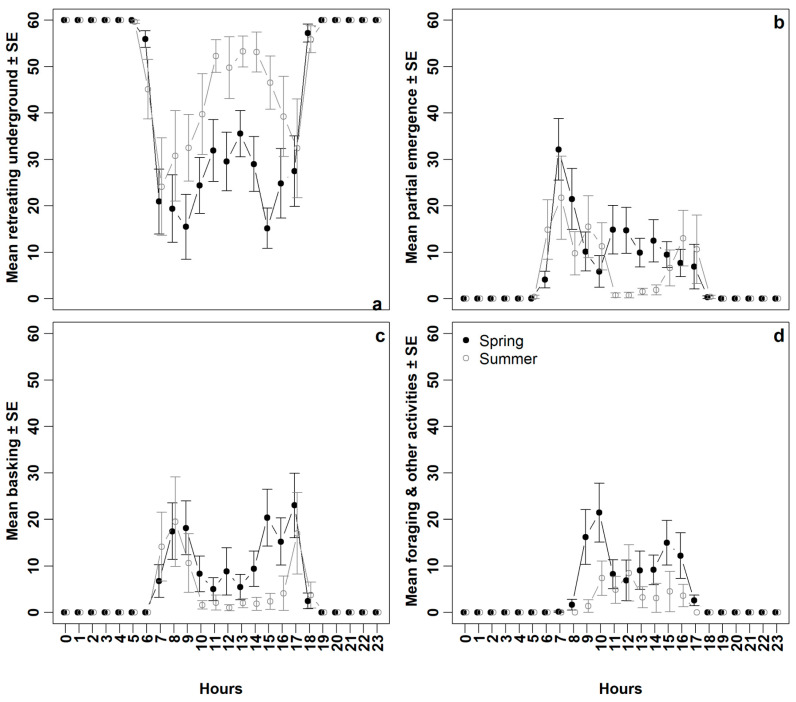
Variations in (**a**) retreating underground, (**b**) partial emergence, (**c**) basking, and (**d**) foraging and other activities between spring and summer (mean ± SE for each hour). Data were collected from 21 individuals, with 13 recorded in spring and 8 in summer.

Spiny-tailed Lizards spent an average of 72.69 ± 2.55% of the day retreating underground in their burrows during spring (*n* = 13 individuals). This increased to 84.32 ± 2.30% in summer (*n* = 8 individuals) (Figure 3a; Table 1 and Table 2). In spring, daily activities –which only occur during the daylight hours—accounted for about a quarter of the day, distributed as follows: 10.42 ± 1.57% for partial emergence, 9.75 ± 1.68% for basking, and 7.14 ± 1.08% for foraging and other activities (*n* = 13 individuals) (Table 1). In contrast, during summer, these activities represented less than a quarter of the day, with 7.57 ± 1.78% for partial emergence, 5.56 ± 1.23% for basking, and 2.54 ± 0.45% for foraging and other activities (*n* = 8 individuals) (Table 1).

### 3.3. Partial Emergence

Spiny-tailed Lizards partially emerged from their burrows primarily in the early morning, with a peak during the hour between 7:00 and 7:59 (Figure 3b). During this hour, they spent an average of 32.15 ± 6.62 min peering out from the burrow entrances in spring (*n* = 13 individuals), compared to 21.75 ± 8.96 min in summer (*n* = 8 individuals), before moving to bask at or near the burrow entrance. The mean soil temperature during this hour was 25.77 ± 0.61 °C in spring (*n* = 13 days) and 32.14 ± 0.55 °C in summer (*n* = 8 days) (Figure 2). Generally, Spiny-tailed Lizards spent more time peering out from burrow entrances in spring compared to summer (Figure 3b; Table 1 and Table 2). Moreover, in summer, this behaviour shifted to earlier in the morning and did not exceed two minutes during the consecutive hottest hours (11:00–13:59) (Figure 3b).

### 3.4. Basking

Spiny-tailed Lizards had two obvious basking periods in both spring and summer (Figure 3c). However, they devoted more time to basking in spring compared to summer (Figure 3c, Table 1 and Table 2). In spring, the first period occurred during the two hours from 8:00 to 9:59, and the second period during the consecutive hours from 15:00 to 17:59 (Figure 3c). The highest peak in the mooring was during the hour 9:00 to 9:59 (23.00 ± 6.93 min, n = 13 individuals), whilst the highest peak in the evening was during the hour 17:00 to 17:59 (29.58 ± 8.57 min, *n* = 13 individuals) (Figure 3c). The mean soil temperatures during these periods were 37.31 ± 1.12 °C and 32.22 ± 1.07 °C, respectively (*n* = 13 days) (Figure 2). In contrast, in summer the first period where during the consecutive hours from 7:00 to 9:59, with the peak basking time during the hour 8:00 to 8:59 (19.50 ± 9.59 min, *n* = 8 individuals). The mean soil temperature during this hour was 38.98 ± 0.97 °C (*n* = 8 days) (Figure 2). The second basking period occurred during the hour from 17:00 to 17:59 (17.00 ± 8.71 min, *n* = 8 individuals) (Figure 3c), where the mean soil temperature was 41.53 ± 0.42 °C (*n* = 8 days) (Figure 2).

### 3.5. Foraging and Other Activities

Spiny-tailed Lizards spent more time foraging and doing other activities far away from their burrows in spring compared to summer (Figure 3d, Table 1 and Table 2). Two distinct periods were observed in spring: the first during the two hours from 09:00 to 10:59, and the second during the consecutive hours from 14:00 to 16:59 (Figure 3d). The highest peak in the morning was during the hour from 10:00 to 10:59, 21.46 ± 6.29 min (*n* = 13 individuals) (Figure 3d), where the mean soil temperature during this hour was 40.86 ± 1.89 °C (*n* = 13 days) (Figure 2). In the evening, the highest peak in activity was observed during the hour (15:00–15:59, 15.00 ± 4.79 min, *n* = 13 individuals) (Figure 3d), where the mean soil temperature during this hour was 41.79 ± 1.50 °C (*n* = 13 days) (Figure 2). In contrast, during summer there was no obvious pattern, and the lizards did not spend more than five minutes away from their burrows except during the two hours from 10:00 to 10:59 and 12:00 to 12:59, where they spent 7.38 ± 3.70 min and 8.50 ± 6.08 min respectively (*n* = 8 individuals) (Figure 3d). The means soil temperature during these two hours were 47.33 ± 0.85 °C and 53.12 ± 0.86 °C, respectively (*n* = 8 days) (Figure 2).

### 3.6. Brumation

Trail cameras did not capture any daily activity during the autumn and winter seasons; however, lizard tracks were found at burrow entrances in all months of these seasons except January, when no traces were observed. Lizards were seen at or near burrow entrances on days with a clear sky and ground surface temperatures exceeding 35 °C, specifically in September (on two occasions), November (one occasion), December (two occasions), and February (two occasions). These observations suggest that the Spiny-tailed Lizard does not maintain regular daily activity during autumn and winter but becomes active only under favourable temperature conditions. The absence of tracks and sightings in January indicates that the lizard enters a state of complete brumation during this month.

### 3.7. Disturbance

The cameras did not record any predators attacking the lizards during the course of the study. However, in spring, some disturbances caused by livestock and vehicles were observed, with each disturbance lasting an average of 33.25 ± 13.17 min (n = 8 occasions) (Table 3). Upon disturbance, the lizards retreated underground in the burrow and re-emerged partially after 42.63 ± 17.70 min (n = 8 occasions) (Table 3). During the summer, livestock disturbances affected two burrows on two separate occasions. The first disturbance lasted 10 min, while the second lasted 135 min. The affected individuals partially re-emerged after 346 min and 151 min, respectively (Table 3). The cameras also captured a Long-legged Buzzard (*Buteo rufinus*) landing approximately three meters from a burrow entrance. Although the buzzard was in close proximity, it did not pose a threat. The lizard retreated into the burrow but partially emerged again after three minutes (Table 3).

## 4. Discussion

The results of this study showed that soil temperature significantly influenced the daily and seasonal activity patterns of the Spiny-tailed Lizards, with a clear trend of increased activity in spring compared to summer. This finding is consistent with the observations of seasonal variations in this lizard’s activity patterns in response to ambient temperature changes [5,14]. As ectotherms, Spiny-tailed Lizards rely heavily on external temperatures to regulate their activity levels [5,28,29]. Spiny-tailed Lizards show an impressive ability to survive such conditions by mainly adjusting their behaviours to optimize thermoregulation and survival [5,14,30]. These behavioural adjustments such as modifying activity periods, shuttling between shade and sunlight, altering coloration, and retreating to burrows during extreme heat are crucial for their survival [9,20].

The results of this study indicated that Spiny-tailed Lizards spent less than a third of their time outside the burrows in both seasons. This suggests that the burrow system is crucial for their thermoregulation and survival, serving as a refuge and shelter from adverse thermal and weather conditions, as well as protection from predators [24,31]. Spiny-tailed Lizards typically forage and stay within the range of their burrows, though this behaviour can vary depending on the availability of food [20]. Our analysis reveals that Spiny-tailed Lizards spent more time in their burrows during summer than in spring (Figure 3a). Staying/retreating underground behaviour during daylight, mostly during the hottest period of the day, is a strategy employed by Spiny-tailed Lizards to regulate their body temperature and prevent exceeding lethal limits [5,21]. Wilms et al. [31] noted that the burrows of this species retain temperature and humidity very effectively. Cunningham [24] found that at a depth of 30 cm below the entrance of the burrows, the temperature was on average 6 °C lower than the ambient temperature. It should be noted that in terms of being outside of its burrow, the Spiny-tailed Lizard is noticeably seasonal [1]. Studies on different reptiles’ species have emphasised the importance of the burrows system [32] in relatively similar habitats (e.g., the Turpan Wonder Gecko (*Teratoscincus roborowskii*) [33]; and the Slater’s Skink (*Liopholis slateri*) [34].

Spiny-tailed Lizards were notably cautious when venturing out of their burrows, particularly during the early morning in both seasons. They often spent a significant amount of time in partial emergence, where they would cautiously extend just their head or both their head and forelimbs from the burrow entrance (Figure 1). This partial emergence likely reflects a high vigilance strategy—particularly during the initial emergence in the early morning—allowing the lizards to monitor their surroundings while also engaging in partial basking after spending the night underground. It was clear that the Spiny-tailed Lizard had a prolonged session of partial emergence during spring (Figure 3b), which is likely due to the milder soil temperatures during this time compared to summer (Figure 2). The less monitoring/emergence during the rest of the daylight hours did not primarily indicate that the species abandoned its vigilance attitude. The unfavourable thermal condition along with energy gained from the morning basking session might explain such behaviour; both scenarios indicate temperature-related emergence. Cunningham [14] reported on a positive correlation between the time of emergence and ambient temperature in United Arab Emirates. Overall, the Spiny-tailed Lizard is a susceptible species to its surrounding, and normally, it retreats to the burrows when disturbed/threatened [14].

Two obvious bimodal basking patterns were recorded in both seasons, with individuals generally spending more time basking in spring than in summer (Figure 3c). Seasonality is well-documented for its direct and significant influence on Spiny-tailed Lizards’ activity patterns [1,5,22]. Here, the observed activity pattern of basking is strongly connected with a suitable/unsuitable soil temperature during these hours (Figure 2), and ambient temperature as well. Different studies have reported similar results of basking patterns, particularly in the morning, in relatively similar habitats and climatic conditions. Cunningham [14] reported that this species typically basks in the morning during both summer and spring, with a shorter basking session in the afternoon. The influence of the ease of the temperature on basking behaviour patterns can be seen in cooler months, where it occurs mostly during afternoon hours [5,14]. Additionally, several studies have indicated that basking behaviour in lizards serves not only for thermoregulation but also for scanning the environment for predators and prey [6,10,35,36].

Spiny-tailed Lizards spent more time foraging and engaging in other activities in spring compared to summer. In spring, two distinct activity periods were observed, whereas no clear pattern emerged in summer. The increased foraging and other activities in spring may be linked to more favourable thermal conditions, higher food availability, and mating opportunities, whereas these factors are reduced in the summer season [1,8,14].

We observed that Spiny-tailed Lizards ceased daily activity in winter and entered complete brumation, particularly in January. This finding partially aligns with Cunningham’s study [14] of a population in the Abu Dhabi Emirate, United Arab Emirates, where some individuals remained inactive during winter, as evidenced by partially closed burrows. However, our study indicates that the lizards in Ha’il did not emerge at all in January, suggesting a more prolonged and uninterrupted brumation period. These behavioural differences may stem from climatic variation: the Ha’il region likely experiences colder winter temperatures than Abu Dhabi, which could drive deeper brumation to conserve energy in harsher conditions.

The disturbances recorded in this study were not fully investigated to assess their potential impact on the thermoregulation and activity patterns of spiny-tailed Lizards. However, given the limited food resources in arid habitats, disturbances are likely to affect these activities. Spiny-tailed Lizards showed a higher body condition index in protected areas compared to non-protected ones, likely due to the increased vegetation cover found in protected sites [22]. Future research should focus on examining the effects of disturbances on thermoregulation and activity patterns of spiny-tailed Lizards to better understand these potential impacts.

Our study was conducted ten years ago, and since then, climate conditions—and consequently, the behaviour of this lizard—may have changed. Therefore, we recommend further research and long-term monitoring of their daily and seasonal activity patterns in the Ha’il region and across their distribution range to enhance our understanding of their adaptability to climate fluctuations.

## 5. Conclusions

This study offers a detailed examination of the daily and seasonal activity patterns of the Spiny-tailed Lizard in the Ha’il region of Saudi Arabia, providing a comparative perspective with other populations. The findings reveal that the lizards adjust their activities in response to soil temperature changes, showing increased activity in spring compared to summer. In autumn and winter, they exhibited little to no daily activity and seemed to enter full brumation, especially in January. A deeper understanding of these patterns, along with the influence of abiotic and biotic factors, could improve conservation efforts, especially for the northern population, which has not been extensively studied.

## Figures and Tables

**Table 1:** Variations in the percentage of each variable over the entire day, comparing spring (*n* = 13 individuals) and summer (*n* = 8 individuals).

Variables	Spring			Summer		
	N	Mean	Std. Error	N	Mean	Std. Error
Retreating underground	13	72.69	2.55	8	84.32	2.30
Partial emergence	13	10.42	1.57	8	7.57	1.78
Basking	13	9.75	1.68	8	5.56	1.23
Foraging and other activities	13	7.14	1.08	8	2.54	0.45

**Table 2:** Minimal mixed-effect models of the retreating underground, partial emergence, basking, and foraging and other activities (*n* = 21 individuals: 13 were recorded in spring and 8 were recorded in summer). The *df* values represent the numerator and denominator degrees of freedom, respectively.

Explanatory Variables	Response Variables											
	Retreating Underground			Partial Emergence			Basking			Foraging and Other Activities		
	df	F	*p*	df	F	*p*	df	F	*p*	df	F	*p*
**Temperature**	1.482	73.01	<0.001	1.482	107.95	<0.001	1.482	75.12	<0.001	1.482	93.60	<0.001
**Season (spring & summer)**	1.19	19.62	<0.001	1.19	12.77	<0.001	1.19	12.26	0.002	1.19	27.62	<0.001

**Table 3:** Disturbances recorded by the cameras during the study.

Burrow ID	Start	End	Disturbance Duration	Disturbance Type	Partial Emergence After Disturbance
B1	20 April, 16:22	20 April, 16:32	10 min	Sheep and goats	10 min
B4	26 April, 8:20	26 April, 10:20	120 min	Camels	86 min
	26 April, 16:15	26 April, 16:25	10 min	Camels	2 min
B7	26 April, 11:30	26 April, 12:05	35 min	Camels	7 min
	26 April, 12:40	26 April, 13:10	30 min	Camels and cars	2 min
	26 April, 16:12	26 April, 16:35	23 min	Camels and cars	97 min
B9	27 April, 8:14	27 April, 8:50	36 min	Sheep and goats	15 min
B10	1 May, 10:36	1 May, 10:38	2 min	A car	122 min
B13	23 July, 8:10	23 July, 8:20	10 min	Camels	346 min
B16	5 August, 7:39	5 August, 7:40	1 min	A raptor	3 min
	5 August, 8:10	5 August, 10:25	135 min	Camels and sheep	151 min

## Data Availability

The original contributions presented in this study are included in the article.
